# Zinc Oxide Nanoparticles Promise Anticancer and Antibacterial Activity in Ovarian Cancer

**DOI:** 10.1007/s11095-023-03505-0

**Published:** 2023-04-04

**Authors:** Ahmed Bakr Mousa, Raghda Moawad, Yasmine Abdallah, Mazen Abdel-Rasheed, Azza M. Abdel Zaher

**Affiliations:** 1 Obstetrics and Gynaecology Department, Faculty of Medicine, Minia University, Minia, Egypt; 2 Dairy Department, Faculty of Agriculture, Minia University, Minia, Egypt; 3 Plant Pathology Department, Faculty of Agriculture, Minia University, Minia, Egypt; 4 Reproductive Health Research Department, National Research Centre, 33 El-Buhouth St, Cairo, 12622 Dokki Egypt; 5 Pathology Department, Faculty of Medicine, Minia University, Minia, Egypt

**Keywords:** antibacterial activity, anticancer, low cost, non-toxic, ovarian cancer, zinc oxide nanoparticles

## Abstract

**Background:**

Ovarian cancer is the most lethal cancer in gynaecology. Surgery, chemotherapy, and radiotherapy are the most often used cancer-fighting strategies. Post-surgery infection is fairly prevalent, especially among people with insufficient immunity. Zinc oxide nanoparticles (ZnOnps) have amazing biomedical features as anticancer and antibacterial agents.

**Methods:**

We investigated the behaviour of ZnOnps synthesized by green methods on ovarian cancers using established human ovarian cancer cell lines, besides the antibacterial action toward models of gram + ve and gram -ve bacteria. The cytotoxic effect of ZnOnps was calculated using a Sulforhodamine B (SRB) trial. *Staphylococcus aureus (S. aureus)* and *Escherichia coli* (*E. coli)* were tested as models for gram + ve and gram -ve bacteria. The selected bacteria were subjected to concentrations of 20, 40, 80, and 100 μg/ml.

**Results:**

The synthesized ZnOnps induced 50% inhibitory concentration (IC50) at a concentration of 27.45 μg/ml. The diameter of inhibition ranged between 20.16 ± 0.16 and 27 ± 0.57 mm for *S. aureus* and 25.66 ± 0.33 to 31 ± 0.33 mm for *E. coli*. ZnOnps antagonistic effect statistically differed with neomycin, cefaclor, and cefadroxil.

**Conclusions:**

Green synthesis of ZnOnps is easily prepared, low cost, non-toxic, and eco-friendly. Their cytotoxic action on SKOV3 cells and their antibacterial characteristics pave the way to be an alternative therapy for ovarian cancer and *S. aureus* and *E. coli* infection.

## Introduction

Cancer is the world’s second cause of mortality [1, 2]. Ovarian cancer remains the most fatal of all gynaecological cancers and the most commonly cited reason for cancer-related death among women. According to the American Cancer Society, 19,710 women will be newly diagnosed with ovarian cancer in the United States in 2023. About 13,270 women will lose their lives to ovarian cancer [3]. In Egypt, there is a remarkable rise in figures of incident ovarian cancer patients from 2288 in 2013 to 5957 in 2050, nearly 260% of the 2013 percentage [4, 5]. The high prevalence of drug-resistant recurring tumours and the exceedingly dismal 5-year survival rates emphasize the limitations of existing ovarian cancer therapies [6]. Clinical science faces a challenge in developing new cancer treatments with low side effects, great selectivity, and potency [1, 2].

One of the biggest challenges to modern public health is antimicrobial resistance (AMR). It has been promoted by increased exposure and the misuse of antibiotics [7, 8]. According to the UK Government-commissioned Review on AMR, it might kill 10 million people annually by 2050. Africa’s low-income countries have the greatest global death rate from AMR [9]. AMR had a role in an estimated 495 million deaths in 2019, of which 127 million were directly related to the disease.

As a result, we have had to explore designing safe, effective, and environmentally friendly antimicrobial agents [10, 11]. Secondary bacterial infections might increase the risk of death in intensive care units; particularly, bacterial coinfection and secondary infection have been reported in COVID-19 patients [12].

The annual incidence of *Staphylococcus aureus (S. aureus)* infection varies between 1 to 3 cases per 10,000 people [13]. *S. aureus* is a prevalent human pathogen accountable for local infections. *S. aureus* has proved to be resistant to commonly used antimicrobial agents, e.g., penicillin, methicillin, tetracycline, erythromycin, and vancomycin [14]. Most pathogenic bacteria are capable of developing resistance to at least some antibiotics [15]. Microbial resistance to antibiotics is acquired through a variety of mechanisms, including drug penetration prevention [16], alternations in the antibiotic target [17], antibiotic enzymatic inactivation, and active antibiotic excretion from a cell [18].

Infections are one of the most frequent complications in patients with cancer [19]. A patient with cancer is more likely to die from a fatal infection than a patient without cancer. It is believed that the primary or contributing factor in the death of about 50% of people with solid haematological malignancies [20].

The most frequent cause of infections in cancer patients is bacteria. Antibiotic failure increases the risk of sepsis, sepsis-related death, and sepsis-related care costs in cancer patients. According to a recent survey conducted in the United Kingdom, 46% of oncologists are concerned that drug-resistant infections may make chemotherapy problematic as a cancer treatment [21].

Green synthesis is defined as using environmentally compatible materials such as bacteria, fungi, and plants to synthesize nanoparticles [22]. Zinc oxide nanoparticles (ZnOnps) instilled by “green synthesis” have antibacterial action against gram -ve and gram + ve bacteria and several Candida fungi [23]. Utilizing metal nanoparticles and their oxides is one of the potential strategies to overcome antibiotic resistance [12]. ZnOnps is easily prepared, not expensive, and safe [24]. It has been used in different biomedical sectors like medical equipment, theranostics, tissue engineering, health care, drug delivery, anticancer, antimicrobial, and antidiabetic [25, 26].

The mode of action of ZnOnps as an anticancer and antibacterial can be explained by metal oxide nanoparticles interacting with bacteria or cells, producing reactive oxygen species (ROS). By assaulting the cell membrane and then attaching to the -SH group of cellular enzymes, the respiratory chain is impacted by the metal ions the nanoparticles create, and certain enzymes are blocked as a result. Therefore, singlet oxygen, hydroxyl radicals, hydrogen peroxide, superoxide anions, and other ROS are created and start to build up. ROS can harm the proteins and DNA found inside bacteria [6, 15, 16, 27].

The Organization for Economic Cooperation and Development guides the health hazards of ZnO NPs/MPs based on their unique physicochemical properties obtained through synthesis procedures [28]. The US Food and Drug Administration has certified the use of ZnO as “generally recognized safe”. As a result, ZnO NPs/MPs have a promising future in biomedical applications such as pharmaceuticals, medical equipment, and cosmeceuticals that are linked to antibacterial properties that overcome drug resistance [23].

The study goal is to investigate the cytotoxic effect of ZnOnps, made through a green synthesis, on ovarian cancer, as well as their antimicrobial activity against gram + ve and gram -ve bacteria, to provide a new treatment option for both cases.

## Materials and Methods

### Preparation of ZnOnps

**Fig. 1:**
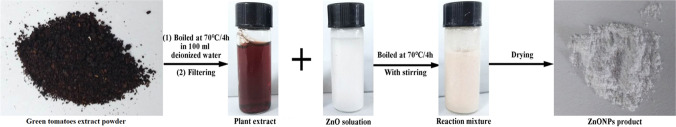
Schematic Presentation of Green Synthesis of ZnOnps using Green Tomatoes Extract.

ZnOnps were obtained through the green synthesis method using green tomato extract (Fig. 1), as previously synthesized and characterized through the biological technique explained by Abdallah *et al*. [29, 30]. Cultivated fresh green tomatoes identified as Lycopersicon esculentum (Solanaceae) were purchased from a local market and used for the synthesis of ZnOnps, in compliance with the relevant institutional, national, and international guidelines and legislation. Tomatoes were dried and grinded. Two grams of dried green tomatoes were placed in a 250 mL flask containing 200 mL of deionized water and then boiled in a 60º C water bath for 4 h. After filtering twice with filter paper Whatman no. 1, the aqueous green tomato extract was added to 100 mL of 1 M ZnO (Sangon Biotech Co., Ltd., Shanghai, China) directly to the installation ZnOnps [29, 31]. The nanoparticles were centrifuged at 10,000 g for 20 min. The supernatant was discarded, and the nanoparticle pellet was washed with distilled water. The nanoparticle powder was obtained by freeze-drying in ALPHA 1–2/LD-Plus vacuum for 8 h. Then ZnOnps characterized UV–Vis spectroscopy (Shimadzu spectrometer, Japan), Transmission Electron Microscopy (TEM) (JEM-1230, JEOL, Akishima, Japan), Scanning electron Microscopy (SEM) (TM-1000, Hitachi, Japan) Fourier transform infrared spectroscopy (FTIR) (Vector 22, Bruker, Germany) and X-ray diffraction (XRD).

### Cell Culture

SKOV3: Ovarian cancer was obtained from Nawah Scientific Inc., Mokatam, Cairo, Egypt. Cells were preserved in RPMI media supplemented with 100 mg/mL of streptomycin, 100 units/ml of penicillin, and 10% heat-inactivated fetal bovine serum in humidified, 5% (v/v) CO_2_ atmosphere at 37°C.

### Cytotoxicity Assay

Sulforhodamine B (SRB) assay was used to evaluate the cell viability. In 96-well plates, aliquots of a 100 μL cell suspension (5 × 10^3^ cells) were incubated in full medium for 24 h. Cells were then treated with a second portion of 100 μL medium containing drugs at varying concentrations. Cells were fixed by replacing the medium with 150 μL of 10% TCA and incubated at 4° C for 1 h after 72 h of drug exposure. After removing the TCA solution, the cells were washed five times with distilled water. Aliquots of 70 μL SRB solution (0.4%w/v) were added and incubated in a dark place at room temperature for 10 min. Plates were washed three times with 1% acetic acid and allowed to air-dry overnight. Then, 150 μL of TRIS (10 mM) was added to dissolve the protein-bound SRB stain; the absorbance was measured at 540 nm using a BMGLABTECH®- FLUOstar Omega microplate reader (Ortenberg, Germany) [32].

### Bacterial Strains

Pathogenic bacteria: *Staphylococcus aureus (S. aureus)* and *Escherichia coli* (*E. coli)* were obtained from the Microbiology Department, Faculty of Agriculture, Minia University.

### Antibacterial Assay

The biological activity of the prepared ZnOnps was tested against *Staphylococcus aureus* and *Escherichia coli* as models for gram + ve and gram -ve bacteria, and zinc oxide (ZnO) was used as a control. The agar well diffusion technique method by Lorentz *et al*. was employed to investigate the antibacterial activity of the ZnOnps [33]. Using a nutrient agar medium, the Petri plates were prepared. The bacteria were extended on nutrient agar to obtain their strain. The well was 6 mm in diameter and contained samples of four different concentrations: 30 μl (20 μg/ml), (40 μg/ml) and (80 μg/ml) (100 μg/ml). The inoculum was spread on the surface of the agar and incubated overnight (16–18 h) at 37 ◦C. The zone of inhibition developed around the well was measured in millimetres.

To assess the performance of ZnOnps, our antibiotics novobiocin & cefadroxil (Oxoide, England) and neomycin & cefaclor (Bioanalysis, Turkey) at a concentration of 30 μg were tested against the same strains by using disk the diffusion method [34].

### Statistical Analysis

Every assessment was performed at least three times. Means and standard deviations were calculated. Data were analyzed by one-way ANOVA analysis followed by the Tukey test for multiple comparisons to determine the differences between groups using GraphPad Prism analysis software.

## Results

**Fig. 2:**
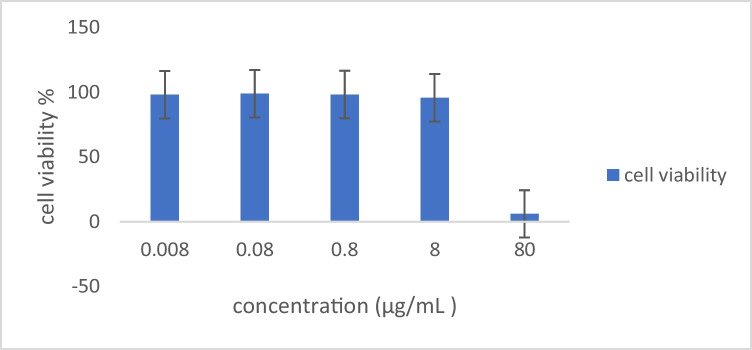
Viability of SKOV3 Cells against ZnOnps and IC50 Calculation.

The cytotoxicity of ZnOnps was investigated using SRB against the SKOV3 cell line that was cultured in 96 wells plates. Various concentrations of (0.008,0.08,0.80, 8 and 80) μg/mL of the ZnOnps were used in the cell lines and tested after being incubated for 72 h at 37◦ C in 5% CO2. 50% inhibitory concentration (IC50) was noted at various ZnOnps concentrations. The cytotoxic activity of ZnOnps was determined by an SRB assay by calculating a cell viability percentage. The synthesized ZnOnps demonstrated concentration-dependent cytotoxic behaviour in ovarian cancer cells. Phyto-synthesized ZnOnps have caused IC50, at a concentration of 27.45 μg/ml, of anticancer activity to SKOV3-cells. Figure 2 indicates the concentration of the ZnOnps against the percentage of SKOV3 in this sample. We used the following equation for estimation:

Viability% = OD sample/ OD control × 100.

In light of ZnOnps having a potential antibacterial action, *Staphylococcus aureus* and *Escherichia coli* were selected as gram + ve and gram -ve bacteria models, respectively.

**Fig. 3:**
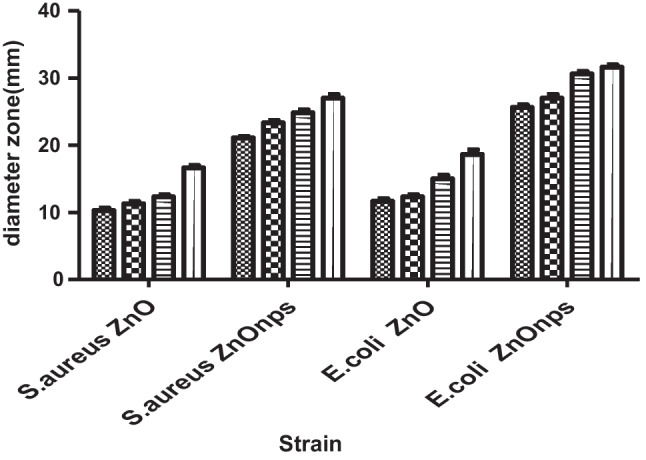
ZnO and ZnOnps Activity Against *S. aureus* and *E. coli* as Inhibition Diameter Zone in mm Inclusion Diameter of the Well (6 mm).

**Fig. 4:**
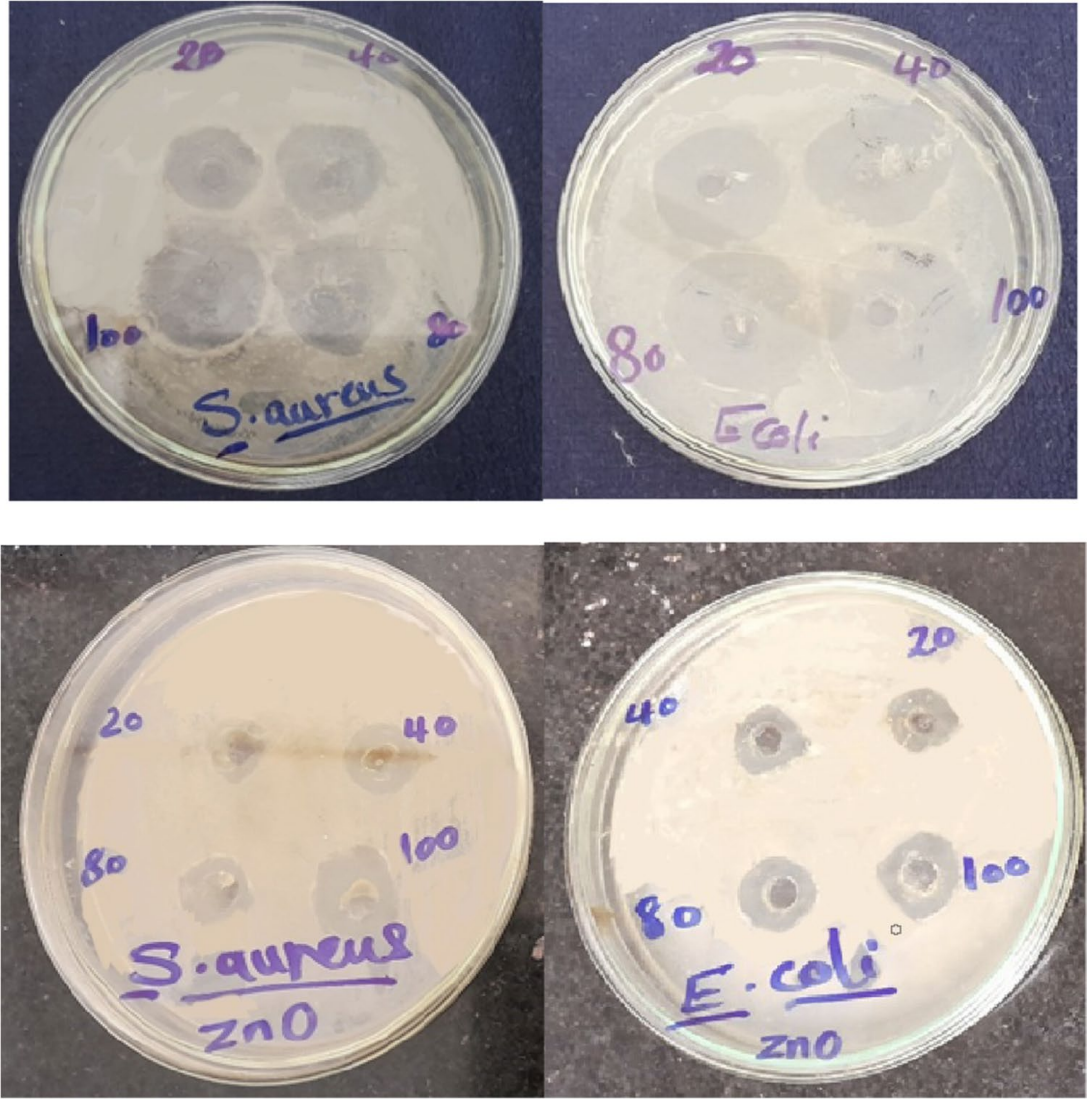
Antimicrobial Activity of ZnOnps and ZnO Against *S. aureus* and *E. coli.*

Concentrations of 20, 40, 80 and 100 μg/ml were attributed to bacterial growth inhibition (Figs. 3 and 4). The inhibition zone surrounding each well was used to measure antibacterial activity, as shown in Fig. 4. Depending on the kind of microorganism, the antibacterial efficiency varied; nonetheless, high inhibition zones were recorded for each of the four ZnOnps doses utilized in our study. For *S. aureus,* the diameter of inhibition ranged between 20.16 ± 0.16 and 27 ± 0.57 mm. The inhibition zone diameter expanded with the gram -ve bacteria, in which the inhibition zone ranged from 25.66 ± 0.33 to 31 ± 0.33 mm. Furthermore, our findings revealed that biosynthesized ZnOnps showed a strong antibacterial impact on the microorganisms examined.

**Fig. 5:**
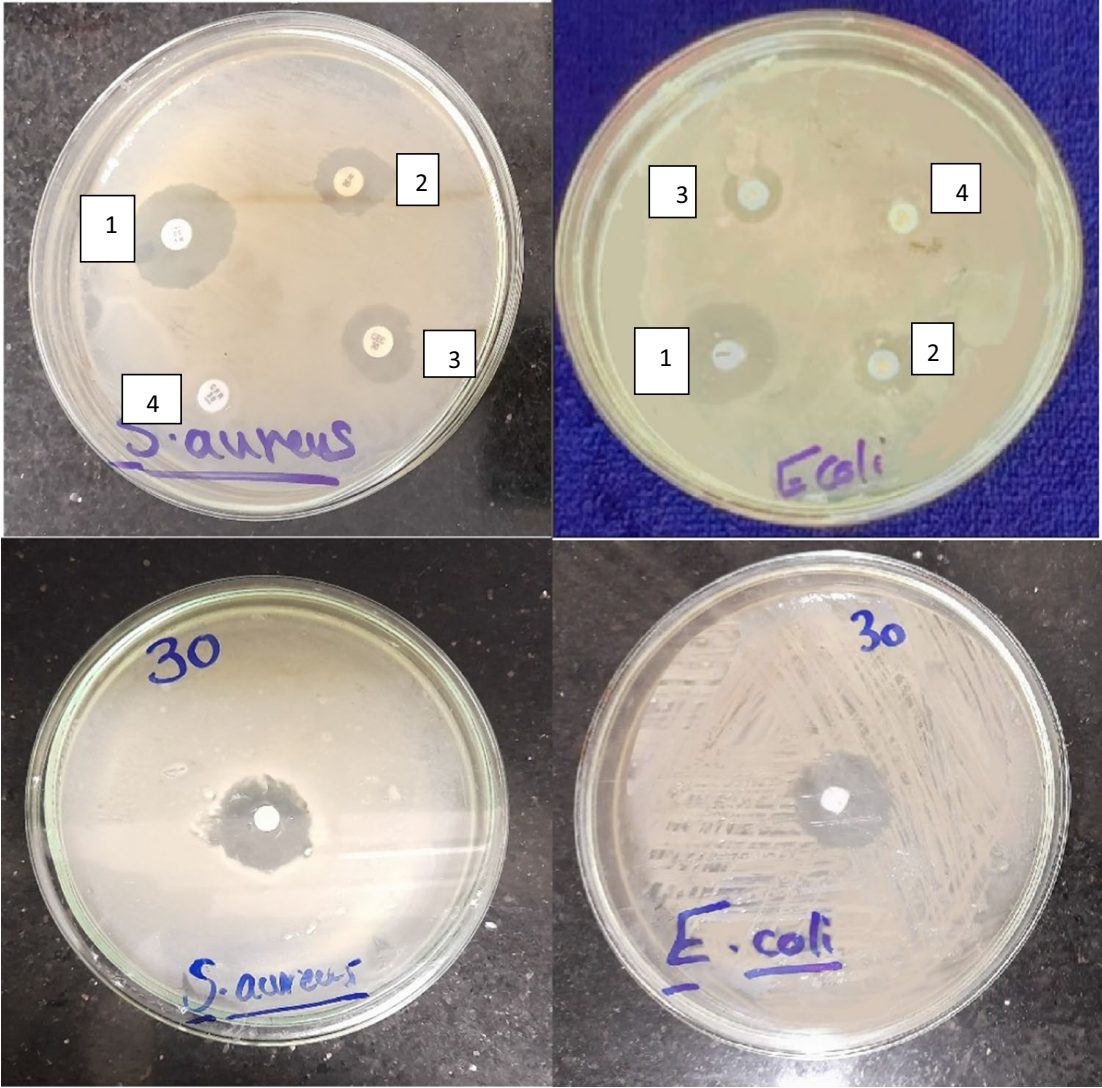
Antimicrobial Activity of Four Antibiotics (1-novobiocin, 2-neomycin, 3-cefaclor and 4-cefadroxil) and ZnOnps against *S. aureus* and *E. coli.*

**Fig. 6:**
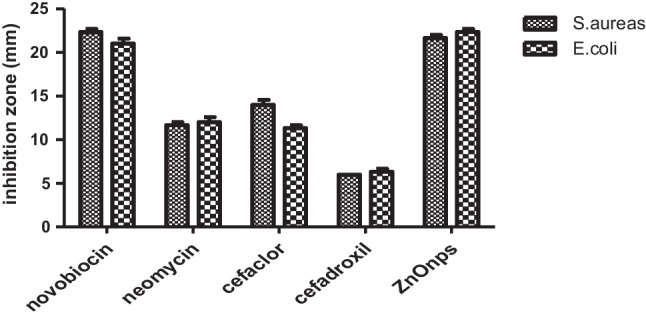
Antimicrobial Activity of Four Antibiotics (novobiocin, neomycin, cefaclor and cefadroxil) and ZnOnps Against *S. aureus* and *E. coli.*

To evaluate the antibacterial activity of ZnOnps, four antibiotics at a concentration of 30 μg were tested for their activity against *Staphylococcus aureus* and *Escherichia coli* using the disk diffusion method (Fig. 5). Results revealed that the two strains were sensitive to novobiocin with inhibition zones 22.3 ± 0.58 and 21 ± 0.58 mm, moderately sensitive to neomycin and cefaclor 1.67 ± 0.33, 12 ± 0.58 mm and 14 ± 0.58, 11.33 ± 0.33 mm, respectively and resistant to cefadroxil. However, ZnOnps appeared to have a slightly better antagonistic effect on the selected stains 21.67 ± 0.33 and 22.33 ± 0.33 mm. ZnOnps was statistically different with neomycin, cefaclor, and cefadroxil (Fig. 6).

## Discussion

The current innovation relates to green metal nanoparticle production, specifically the synthesis of Zinc nanoparticles employing tomato extracts as a reducing and capping agent. Nanotechnology, which involves producing various copper, magnesium, gold, and silver, among other metals, has all been used to create similar structures. Because of their remarkable physiochemical properties, such as their huge surface area, drug delivery capabilities, and low toxicity, nanoparticles have gotten a lot of interest. Because it is abundantly available, cost-efficient, and has no known adverse effects, biological nanomaterial synthesis is an environmentally beneficial technology. Due to their antimicrobial properties, NPS can be used in various applications, such as foods, pharmaceuticals, sunscreens, and cosmetics. As a result, a method of generating metal nanoparticles using tomato extract is desired, as it solves the concerns mentioned above [35].

Recent studies have shown that ZnOnps exhibit a high degree of cancer cell selectivity with the ability to surpass the therapeutic indices of some commonly used chemotherapeutic agents *in vivo* studies [36]. Although ZnOnps are considered generally recorded as safe (GRAS), the behaviour of metal-oxide nanoparticles are varied from normal to a cancer cell. It can be summarised as follow after the dissociation process, metal ion enters a normal cell and acts as a supplement or inhibits cancer cell by metal cation homeostasis; however, the other part (oxygen radicals) turns into antioxidants or ROS, respectively [37]. The cytotoxic effect of ZnOnps, made through a green synthesis, has to be investigated *in vitro* and *in vivo* to role out the safety and possible systemic use.

Bai *et al*. indicated that cell viability was significantly reduced after the application of 20 and 30 μg/mL ZnOnps (*P* < 0.05) [38]. Although there were substantial variations in cell viability between 10 and 30 μg/mL, a notable difference was detected between 20 and 30 μg/mL. Human ovarian cancer cell viability reduced considerably in a time- and dose-dependent manner after 24 h of exposure to 20 and 30 μg/mL ZnOnps (*P* < 0.05). There was no significant variation in cell viability between 12 and 24 h after exposure to a lower concentration of 5 and 10 μg/mL. Overall, a significant effect was detected at greater doses of 20 and 30 μg/mL after 24 h; nevertheless, a substantial change in cell viability was observed when the concentration increased from 20 to 30 μg/mL. As a result, 20 and 30 g/mL are likely feasible doses for determining the impact of ZnOnps on ovarian cancer cells.

Lee *et al*. demonstrated the ZnOnps toxicity on human epidermal keratinocyte HaCaT cells after 24 h of exposure to concentrations of 0,10,20,40 and 80 μg/mL [39]. The results showed that ZnOnps impaired mitochondrial function and caused LDH leakage. Furthermore, ZnOnps caused oxidative stress, resulting in ROS and LPO production. A concentration-dependent impact was seen in the human lung adenocarcinoma cell line LTEP-a-2 [40], which was consistent with our findings. Chuang *et al*. showed how human coronary artery endothelial cells responded to two distinct sizes of ZnOnps, such as 20 and 90 nm [41].

In the present study, it is realized that the ZnOnps with the size of 51.6 ± 3.6 nm have slight cytotoxicity at concentrations up to 8 μg/mL around 95.6 ± 2.04 to 98.8 ± 1.27% determined as viability percentage. However, at higher concentrations, significant toxicity has been observed. At 80 μg/ml, only 5.99 ± 1.60% of the cancer cells were still viable, and the IC50 was at a concentration of 27.45 μg/ml. These results are consistent with Gu *et al*. study [42], where the IC50 was (27.45 μg/ml) for 24 h of incubation. The duration variation of incubation may be due to using synthesized ZnOnps compared to our naturally derived ones. On the other hand, our results are extremely different from Alipour *et al*. [43]. They studied the cytotoxic effect of ZnOnps on SKOV3 cell lines, and the IC50 was at a concentration of 8.05 μM at 72 h. This difference may be due to using a different assay for cytotoxicity evaluation (MTT assay) while we used SRB assay, and also, the naturally derived ZnOnps may have a role. Their results showed that ZnOnps significantly increased the response to Cisplatin in SKOV3 cells. From the present study, it can be concluded that the naturally-derived ZnOnps are a promising tool and can be used as an anticancer agent with high effectiveness in ovarian cancer. ZnOnps had a distinct cytotoxic impact on SKOV3 cells, as well as a clear concentration–response association (Fig. 2).

Production of ROS has previously been demonstrated to be a characteristic of ZnOnps [44–46]. The antioxidative ability of cells can be depleted by high and persistent ROS build-up, resulting in cell death. Cancer cells have greater cellular ROS levels than healthy cells due to their increased metabolic needs and quick growth rate. The therapy with ZnOnps is particularly effective against cancer cells because of their innately higher oxidative state. Proteins may get ubiquitinated due to oxidative damage brought on by high ROS build-up. Proteotoxic stress results from the increased burden on the protein degradation machinery caused by the accumulation of ubiquitinated proteins in cells. Additionally, Padmanabhan *et al*. discovered that the ZnOnps treatment reduced the antioxidative ability of ovarian cancer cells and increased proteotoxic stress [6].

Continuous breakthroughs in green nanotechnology, as well as the encouraging findings of this study, point to the use of rod-shaped nanostructures for therapeutic purposes [6]. Previous research showed that the ZnOnps cause ovarian cancer cells to experience acute oxidative and proteotoxic stress, which causes the cells to undergo apoptosis and death [46]. The generation of ROS through oxidative stress is the main probable mechanism of NPs exposed toxicity. Elevated ROS serves as apoptotic stimuli. ZnO-NPs cause a significant increase in the expression of cell cycle checkpoint proteins p53, bax, and caspase- 3 while down-regulated antiapoptotic bcl-2 protein [47]. Also, It has been documented that ZnO-NPs may induce apoptosis through intrinsic mitochondrial pathways. They decrease mitochondrial membrane potential and, conversely, increase bax/bcl2 ratio, so they induce apoptosis [48]. The green synthesis-produced HA/ZnO nanocomposite has the potential to become a highly effective cancer treatment [49].

Depending on how the cell wall reacts with the nanoparticles, metal oxide nanoparticles generally have a strong antibacterial effect against any type of bacteria, whether gram + ve or gram -ve. However, *Cucumis melo*-assisted ZnOnps demonstrated stronger antibacterial activity against both types of bacteria *(S. aureus and E. coli)*, which focuses on the substance that can penetrate the cell membrane. Among the two types of bacteria, *E. coli* has a thick impermeable cell wall lipopolysaccharide that is not easily destroyed by chemical compounds. However, the generated ZnOnps disrupt this cell membrane and inhibit the growth region, presumably due to the appearance of Zn2 + during surface reactivity or the creation of Reactive Oxygen Species and cell wall destruction. The size of the nanoparticles is also important in blocking the membrane of the bacteria. According to the findings, physical interaction between bacteria and metal nanoparticles initiated antibacterial activity [50]. ZnOnps can connect to negatively charged bacterial cell surfaces via electrostatic contact because of their cation characteristics, which leads to a build-up of ZnOnps on the surface of microorganisms’ cell walls and subsequent cell damage [51].

According to our results, multi-resistant S. aureus was less inhibited than *E.coli* when it was cultivated with 100, 250 and 500 μg/ml of α-Fe2O3@ZnO nanocomposite [52]. In the same manner, Archana *et al*. reported that the inhibition zone observed for *E. coli* was 6.70 ± 0.46, higher than those obtained for *S. aureus,* 6.20 ± 0.43 mm at 100 μg/ml [51]. These results were lower than those obtained in this study, 31 ± 0.33 and 27 ± 0.57 mm for the two tested strains at the same concentration. The variation may be due to the preparation method and the particle size.

Novobiocin’s mode of action is to inhibit DNA gyrase by binding the ATP-binding site in the ATPase subunit. Although novobiocin is generally effective against gram + ve bacteria, it is reported that it can be used for sensitive E.coli that has a leaky outer membrane, as liposaccharides in the outer membrane represent a barrier [53]. This may be explained by the sensitivity of the *E.coli* strain incorporated in this study which appears to have an inhibition zone *of* 21 ± 0.58 mm.

Neomycin belongs to aminoglycosides, which inhibit bacterial protein synthesis. It effectively works gram -ve bacteria and is commonly used for perioperative prophylaxis [54]. Bessa *et al*. (2016) tested 68 *S. aureus* isolates and established that about 57.4% of *S. aureus* were sensitive to neomycin by using the disk diffusion technique. These results confirmed that the tested *S. aureus* was sensitive to neomycin but selectively to gram -ve bacteria [55]. In 2012–2017, neomycin susceptible *E.coli was* estimated to be about 87.15% [56].

Cefaclor, a second-generation cephalosporin, works similarly to penicillins. As a beta-lactam antibiotic, it inhibits the third and last stage of bacterial cell wall synthesis. It acts on both gram + ve and gram -ve bacteria [57]. About 73.6% of *E.coli* isolates from different clinical specimens in children in 2009 were sensitive to cefaclor [58]. Cefadroxil, a first-generation cephalosporin, also works similarly to penicillins. It is more effective for gram + ve [57]. *S. aureus and E.coli* attributed in our work were resistant to cefadroxil.

Many reports discussed antibiotic resistance of *S. aureus* and *E.coli*; 42.6% of *S. aureus* were resistant to neomycin. In 2012–2017, neomycin-resistant *E.coli was* estimated at about 12.85%, concerning cefaclor, 2,6.4% of *E.coli* isolates from different clinical specimens in children in 2009 were resistant [55, 56]. A study examined 100 *E.coli* revealed that 31, 65, 60, 85, 0, 20, 33, 5, 6, and 47% of the strains were resistant to Ceftazidime, Cefuroxime, Ceftriaxone, Amoxicillin, Meropenem, Gentamicin, Ciprofloxacin, Cefoperazone-sulbactam, Amoxicillin-clavulanic acid and Sulphonamides, respectively [59].

Rana *et al*. (2022) studied 92 *S. aureus* and 34 *E. coli* isolates and reported that all the isolates were multi-drug resistant (MDR), resistant to equal or more than three antimicrobial classes. For *S. aureus* isolates, the maximum resistance was recorded for tetracycline (76.09%), oxacillin, and ampicillin (70.65%). More than 58% of the isolates exhibited resistance against erythromycin, amoxicillin-clavulanic acid and streptomycin. Regarding *E. coli,* the highest resistance was observed against oxacillin (64.71%) and ampicillin (58.82%) antibiotics. Oproxamitelly 30% of *E. coli* isolates were resistant to ceftriaxone, ciprofloxacin and cefaclor [60]. That supported the urgent need for exploring alternative antibacterial agents to avoid AMR phenomena. We introduce a green synthesized ZnOnps as a safe, easy-to-make, cost-effective solution for AMR for both gram + ve and gram -ve bacteria.

Krishnamoorthy *et al*. (2022) nominated ZnO NPs to be used as an alternative antibacterial agent against β-lactam-resistant gram-ve pathogenic bacteria [61]. These findings proved the efficacy of green synthesized ZnOnps as a tremendous antibacterial agent. The utilization of 27.45 μg/ml ZnOnps is sufficient to achieve a considerable impact on SKVO3 ovarian cells and both gram + ve and gram -ve bacteria.

However, eco-friendly technologies based on using fungi, algae, and bacteria to synthesize nanostructures are more popular than physical and chemical resources [62], as the physical method takes a lot of time and energy. A simple but costly chemical technique necessitates high-purity chemicals that are pricey and pose contamination risks. The biological synthesis (eco-friendly) doesn’t require expensive chemicals, high temperatures, or a lot of time and is toxic-free and environmentally benign. Plants and microorganisms are commonly used in this method [63, 64].

The systems were studied for these particles in drug delivery applications in terms of hydrogel rheology and polymer crosslinking degree. Farzaneh Sabbagh and colleagues employed acrylamide-based hydrogel drug delivery systems to release acyclovir from magnesium oxide nanocomposite hydrogels [65]. Acyclovir was soaked into the polymer in the studies, and the system was employed for vaginal medication administration and release. The chemical and physical properties of the fortified hydrogels provided an assessed report on the polymer’s morphological structure, swelling performance, gel bonding generation, and physical properties. The drug release in two various media was examined using PBS and SVF aqueous solutions, and the quantity of the released medication was determined using HPLC. The pH sensitivity and *in vitro* drug release of hydrogels were studied at three different pH levels: 4, 6, and 8 [65].

## Conclusion

ZnOnps synthesized from plants may be an essential research topic in the biomedical sector. The green synthesis of ZnOnps using green tomato extract has been highlighted in this study, as it is a simple, rapid, environmentally safe, and relatively low-cost process. This method can help to overcome the limitations of conventional chemical and physical methods.

ZnOnps are considered a novel generation of antimicrobial agents; their wound healing and antibacterial characteristics stimulate their use after surgical procedures. In addition, their cytotoxic action on SKOV3 cells paves the way for a mutant treatment in gynaecological applications.

Our recommendation is to investigate the toxicity of the green synthesized ZnOnps through in -vivo studies on experimental animals to determine the effect of these nanoparticles on normal cells and then decide whether it can be used systemically or only locally as identifying means to reduce unwanted toxicity.

## Data Availability

The datasets used are available from the corresponding author upon reasonable request.
